# Supplementary figures and images for: Development of Forensically Important Sarcophaga peregrina (Diptera: Sarcophagidae) and Intra-Puparial Age Estimation Utilizing Multiple Methods at Constant and Fluctuating Temperatures
Source: Animals (Basel). 2023 May 11;13(10):1607. doi: 10.3390/ani13101607 (PMC10215118; doi:10.3390/ani13101607)

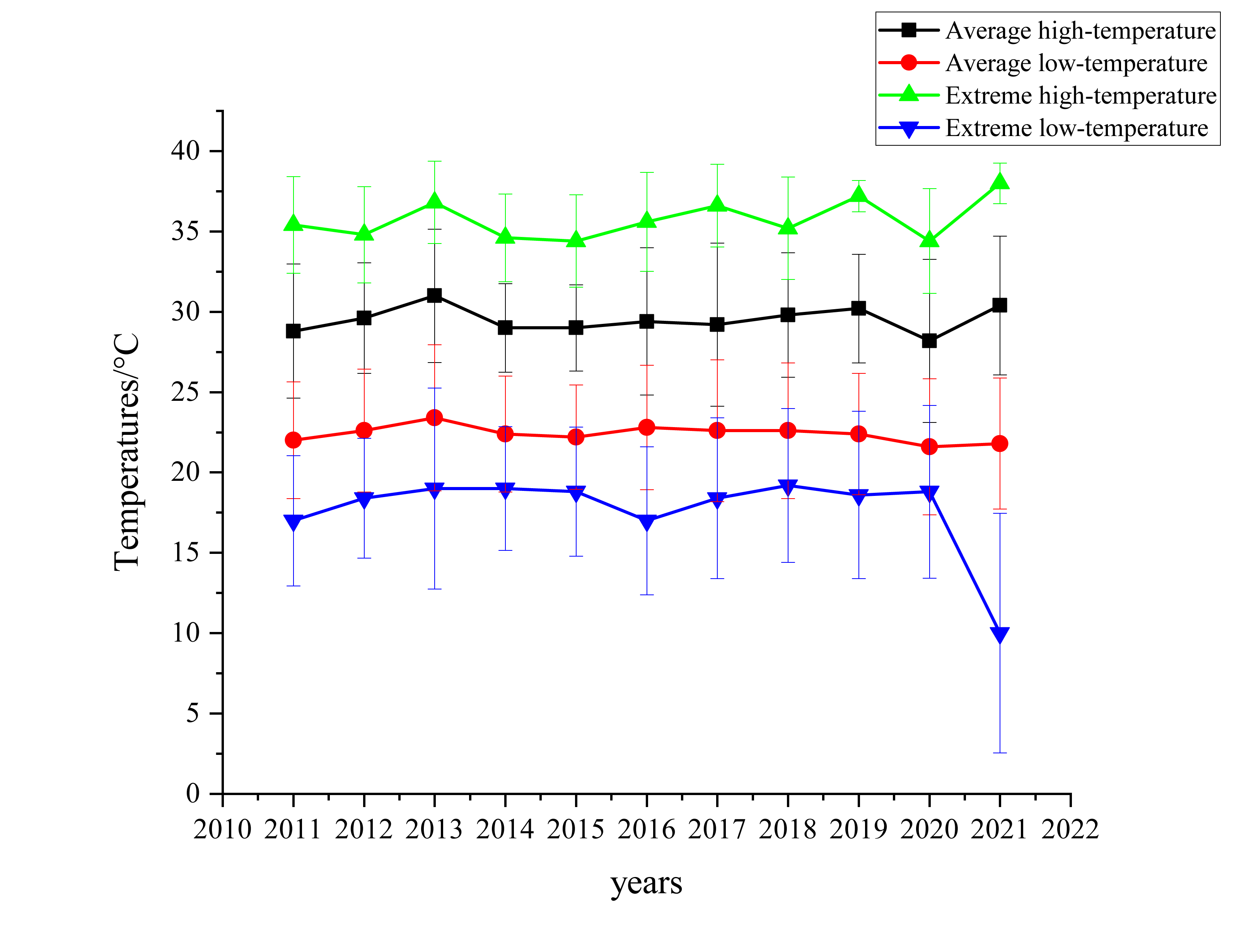

Supplement: Supplementary file 1 [file animals-13-01607-s001.zip › animals-2310679-supplementary/Supplementary/Fig S/Fig S1.tif]

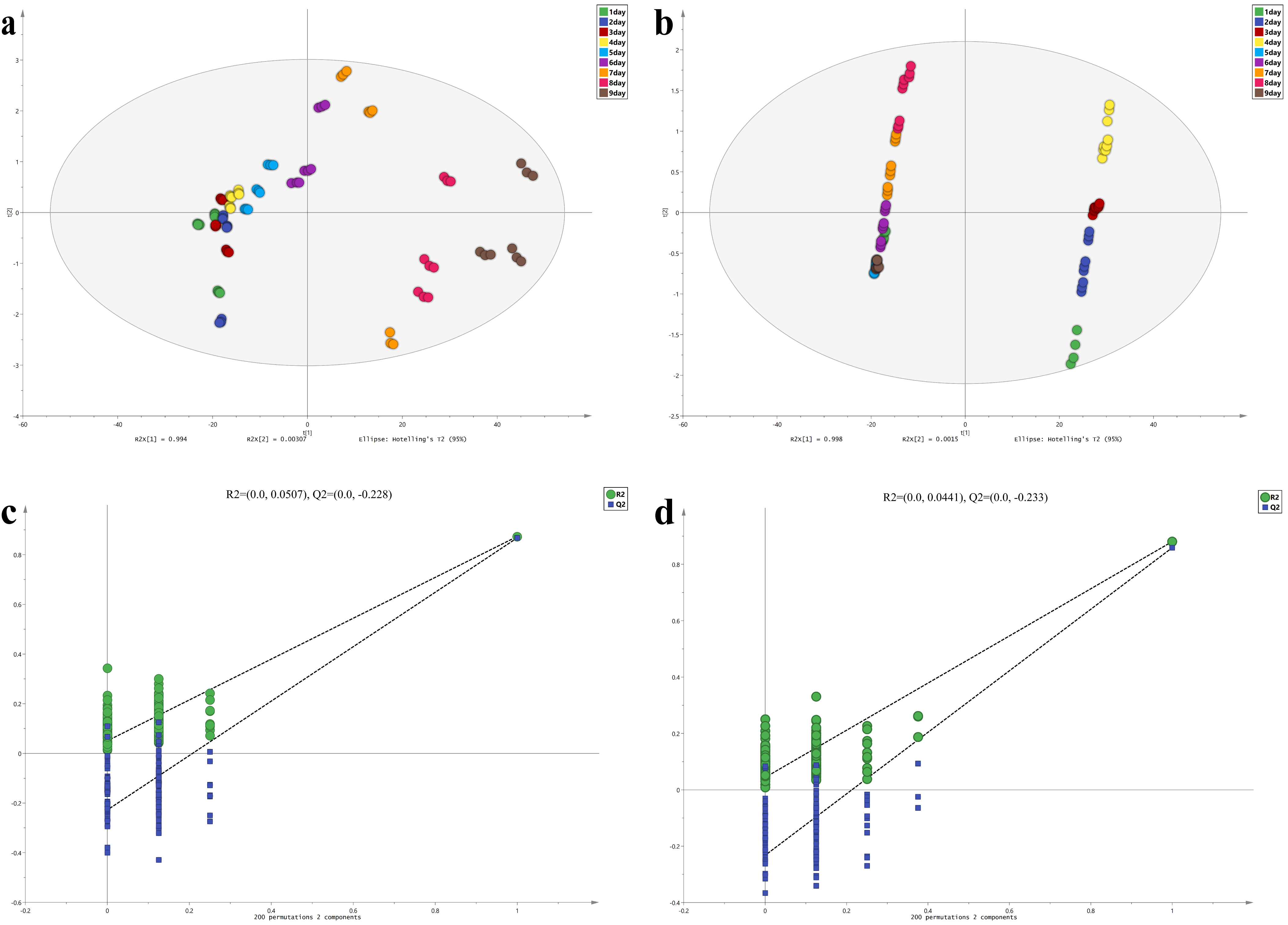

Supplement: Supplementary file 1 [file animals-13-01607-s001.zip › animals-2310679-supplementary/Supplementary/Fig S/Fig S2.tif]

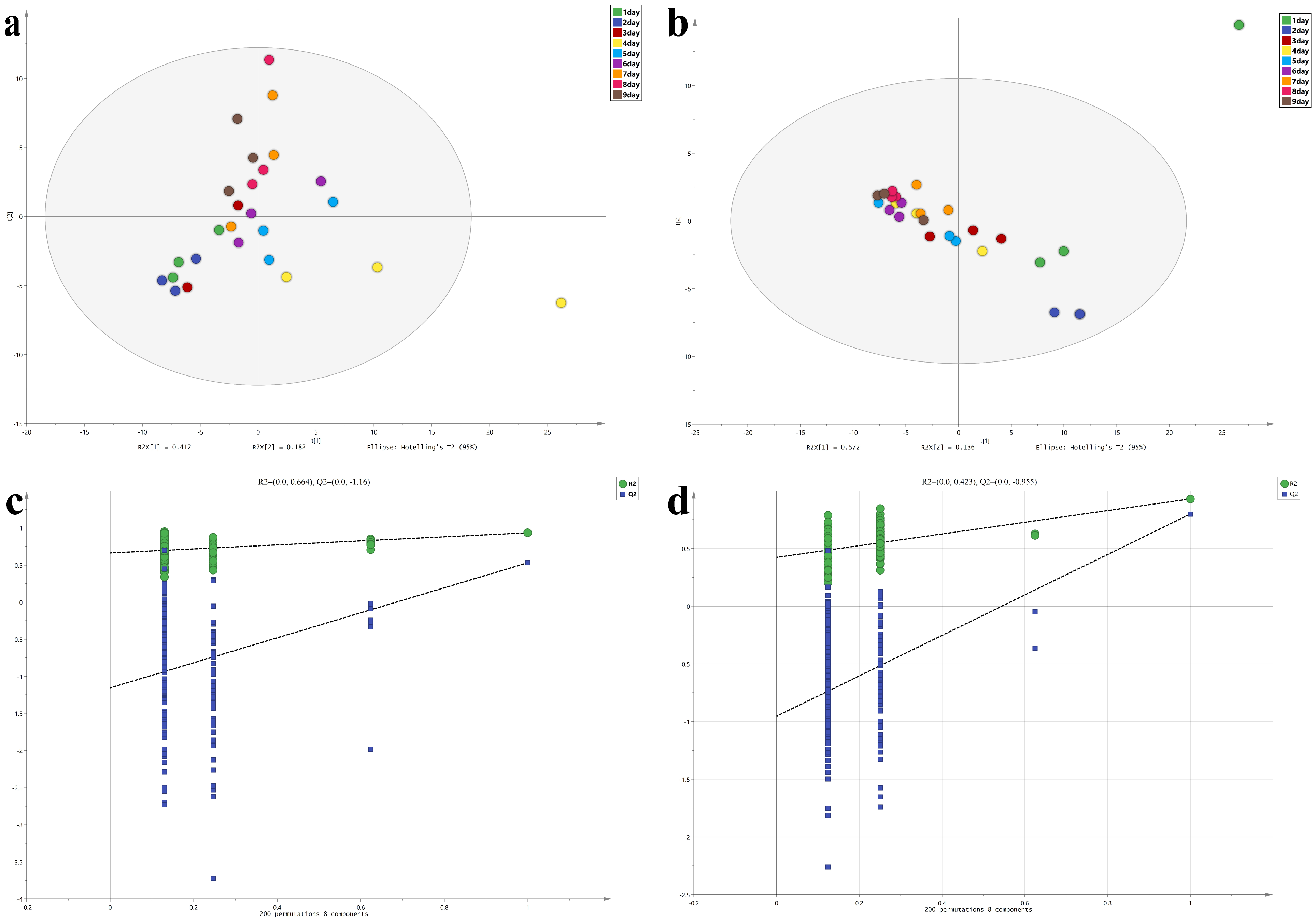

Supplement: Supplementary file 1 [file animals-13-01607-s001.zip › animals-2310679-supplementary/Supplementary/Fig S/Fig S3.tif]
